# Taxonomic study on *Lathrobium* Gravenhorst (Coleoptera, Staphylinidae, Paederinae) from Longwangshan Mountain, East China

**DOI:** 10.3897/zookeys.165.2384

**Published:** 2012-01-13

**Authors:** Zhong Peng, Li-Zhen Li, Mei-Jun Zhao

**Affiliations:** 1 Department of Biology, College of Life and Environmental Sciences, Shanghai Normal University, Shanghai, 200234, P. R. China

**Keywords:** Coleoptera, Staphylinidae, taxonomy, *Lathrobium*, new species, key, Longwangshan, China

## Abstract

Species of the genus *Lathrobium* Gravenhorst from Longwangshan Mountain, Zhejiang, East China are studied. A total of five species are recognized, among which three are described here as new: *Lathrobium lingae* **sp. n.**, *Lathrobium longwangshanense* **sp. n.** and *Lathrobium uncum* **sp. n.**, one species was unidentified and the female of *Lathrobium tianmushanense* Watanabe is newly reported. All of these species are illustrated and keyed.

## Introduction

To the present, a total of 625 species of the genus *Lathrobium* Gravenhorst have been known worldwide, 64 of them from China (Löbl and Smetana 2004 and subsequent papers). Longwangshan Mountain (at. 30°24'N, 119°27'E) in the south of the Anji County forms the Tianmushan mountain range and Mt. West Tianmushan stand 9.5 kilometers apart. Only two species of *Lathrobium* have been recorded by Watanabe (1999) from West Tianmushan Mountain, but no members of the genus have been reported yet from Longwangshan Mountain, Zhejiang Province, East China.

In recent years, we made several collecting trips to the Longwangshan Mountain, and obtained a lot of *Lathrobium* specimens. On the basis of the examination, four species were recognized and one species was unidentified, among which three were revealed to be new, and the female of *Lathrobium tianmushanense* Watanabe was newly discovered. The purpose of this paper is to describe and illustrate the *Lathrobium* species of Longwangshan Mountain, and to provide an identification keyto *Lathrobium* species of Longwangshan Mountain and West Tianmushan Mountain.

## Material and methods

All specimens were collected from the leaf litter of the forest floor by sifting. They were killed with ethylacetate and then dried. Dissections were done in water. The genital organs and other dissected parts were mounted in Euparal (Chroma Gesellschaft Schmidt, Koengen, Germany) on plastic slides that were placed on the same pin as the specimen. Photos were taken by a Canon EOS 40D Camera with an MP–E 65 mm Macro Lens or by a Canon G9 Camera mounted on an Olympus CX31 microscope. The type specimens are deposited in the Insect Collection of Shanghai Normal University (SNUC).

The following abbreviations are used in the text, with all measurements in millimeters:

BL length of the body from the labral anterior margin to the anal end

HL length of the head from the clypeal anterior margin to the head base

HW maximum width of the head

PL length of the pronotum along the midline

PW maximum width of the pronotum

EL length of the elytra from the apex of the scutellum to the elytral posterior margin

## Taxonomy

### Lathrobium lingae sp. n.

urn:lsid:zoobank.org:act:741943E9-A379-42E0-807D-811A596FCE88

http://species-id.net/wiki/Lathrobium_lingae

Figs 1A 3

#### Type locality.

Longwangshan Nature Reserve, Zhejiang Province, East China

#### Type material

(1 ♂). Holotype: ♂, labeled ‘**CHINA:** zhejiang Prov. / Anji County / Longwang Mt. / 25.iv.2006, alt. 950–1,200 m / Rui-Fen Ling leg.’.

#### Description.

Measurements and ratios (holotype):BL 6.88, HL 1.00, HW 1.11, PL 1.27, PW 1.15, EL 1.36, HL/HW 0.91, HW/PW 0.96, HL/PL 0.79, PL/PW 1.11, EL/PL 0.71.

**Figures 1.**
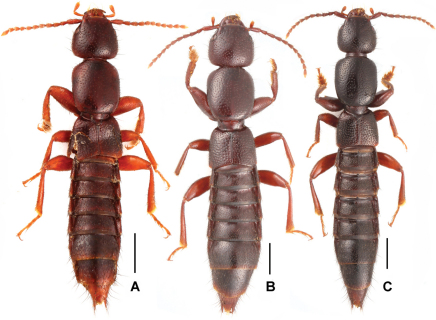
Male habitus of *Lathrobium* spp., **A** *Lathrobium lingae* **B** *Lathrobium longwangshanense* **C** *Lathrobium tianmushanense*. Scales: 1.0 mm.

Male (Fig. 1A). Body brown with paler apex, legs reddish brown, antennae reddish brown to yellowish brown.

Head quadrate; posterior angles broadly rounded; postgenae weakly convex ventrally; integument with coarse and moderately dense punctation; eyes reduced.

Pronotum slightly stocky, slightly broader than head; punctation sparser than that of head; interstices shining, lacking microsculpture.

Elytra at suture distinctly shorter than pronotum; wider than long; punctation well-defined; and hind wings completely reduced.

Abdomen with dense pubescence; sternite VII (Fig. 3A) with short dark modified setae on postero-median semicircular impression; sternite VIII (Fig. 3C) with triangular emargination and with short dark modified setae on deep impression; sternite IX (Fig. 3B) asymmetrical; aedeagus (Fig. 3D, 3E) with conspicuously long, slender ventral process and twisted dorsal sclerites.

Female. Unknown.

#### Distribution.

East China (Zhejiang: Longwangshan Mountain).

#### Etymology.

The species is named after Rui-Fen Ling, who collected the type specimens.

#### Remarks.

The new species is close to *Lathrobium fengae* in similar general form. *Lathrobium lingae* differs especially by the male sternite VII with deeper impression, male sternite VIII with regularly triangular apico-median emargination, and aedeagus with gracile ventral process and single dorsal sclerite. *Lathrobium fengae* has the male sternite VII with shallower impression, male sternite VIII with irregular apico-median emargination, and aedeagus with broad ventral process and two dorsal sclerites.

### Lathrobium longwangshanense sp. n.

urn:lsid:zoobank.org:act:FFAABDE9-CA6B-4DD8-971D-41BC9898C0E0

http://species-id.net/wiki/Lathrobium_longwangshanense

Figs 1B 4

#### Type locality.

Longwangshan Nature Reserve, Zhejiang Province, East China

#### Type material

(1 ♂). Holotype: ♂, labeled ‘**CHINA:** zhejiang Prov. / Anji County / Longwang Mt. / 25.iv.2006, alt. 950–1,200 m / Yong-Yin Wang leg.’.

#### Description.

Measurements and ratios (holotype):BL 9.56, HL 1.51, HW 1.58, PL 1.81, PW 1.59, EL 1.32, HL/HW 0.95, HW/PW 0.95, HL/PL 0.83, PL/PW 1.09, EL/PL 0.73.

Habitus as in Fig. 1B. Externally similar to *Lathrobium lingae*, except for the lighter average coloration, the somewhat larger body size, the denser punctation on the head and the pronotum.

Male. Sternite VI (Fig. 4A) with tufted pubescence same length as concavity; sternite VII (Fig. 4B) with weak emargination; sternite VIII (Fig. 4C) with darkish setae on impression and basal angle of asymmetrical triangular emargination with dense point-like seta; sternite IX (Fig. 4D) slightly acute anteriorly; aedeagus (Fig. 4E, 4F) with distinct long ventral process and twisted dorsal sclerites.

Female. Unknown.

#### Distribution.

East China (Zhejiang: Longwangshan Mountain).

#### Etymology.

The species is named after its type locality.

#### Remarks.

The new species is similar in most respects to *Lathrobium tianmushanense*, but itdiffers in having relatively stout body, HL/PL being more than 0.80, male sternite VI with tufted pubescence at concavity and aedeagus with longer twisted dorsal sclerites. In *Lathrobium tianmushanense*, the body is relatively slender, HL/PL is more than 0.73, the male sternite VI has the concavity lacking pubescence and the dorsal sclerites of the aedeagus are much shorter.

### Lathrobium tianmushanense

Watanabe

http://species-id.net/wiki/Lathrobium_tianmushanense

Figs 1C 5

Lathrobium tianmushanense Watanabe, 1999: 249

#### Type locality.

WestTianmushan Mountain, Zhejiang Province, East China

#### Material studied

(3 ♂♂, 4 ♀♀). 1 ♂, 1 ♀, labelled ‘**CHINA:** zhejiang Prov. / Anji County / Longwang Mt. / Qianmutian / 27.v.2009, alt. 1,300 m, / Yuan, Liu, Feng & Yin leg.’. 1 ♂, 3 ♀♀, same label data, but ‘29.v.2009’.

#### Rescription.

For detailed male description of male see Watanabe (1999: 249).

Female. BL 8.06–8.34; Measurements and ratios: HL 1.17, HW 1.32, PL 1.55, PW 1.36, EL 1.02, HL/HW 0.89, HW/PW 0.97, HL/PL 0.76, PL/PW 1.14, EL/PL 0.88.

Slightly smaller than male; posterior margin of tergite VIII (Fig. 5G) weakly asymmetrical; sternite VIII (Fig. 5H) distinctly pointed in the middle; tergite IX (Fig. 5I) (not separated from X) with long and acute lateral processes; tergite X (Fig. 5I) slightly shorter than tergite IX.

#### Distribution.

East China (Zhejiang: Longwangshan and Tianmushan Mountains).

#### Remarks.

*Lathrobium tianmushanense* is closest to *Lathrobium cooter*i from Zhejiang by sharing a similar general form. It can be readily separated by the male sternite VI with modified setae at the concavity and male sternite VIII with relatively regular emargination. While *Lathrobium cooteri* has the male sternite VI lacking sexual characters and male sternite VIII possess an irregular emargination.

### Lathrobium uncum sp. n.

urn:lsid:zoobank.org:act:FBC72B91-DE34-49D0-9918-A486421C9F48

http://species-id.net/wiki/Lathrobium_uncum

Figs 2A 6

#### Type locality.

Longwangshan Nature Reserve, Zhejiang Province, East China

#### Type material

(7 ♂♂, 5 ♀♀). Holotype: ♂, labeled ‘**CHINA:** zhejiang Prov. / Anji County / Longwang Mt. / 25.iv.2006, alt. 950–1,200 m / Tang Liang leg.’. Paratypes: 7 ♂♂, 5 ♀, same label data as holotype; 1 ♂, 4 ♀♀, same, but ‘Qianmutian / 27.v.2009, alt. 1,300 m/ Yuan, Liu, Feng & Yin leg.’.

#### Description.

Measurements and ratios: BL 5.35–5.93. Holotype: HL 0.78, HW 0.81, PL 1.02, PW 0.82, EL 0.72, HL/HW 0.97, HW/PW 0.98, HL/PL 0.77, PL/PW 1.25, EL/PL 0.70.

**Figures 2.**
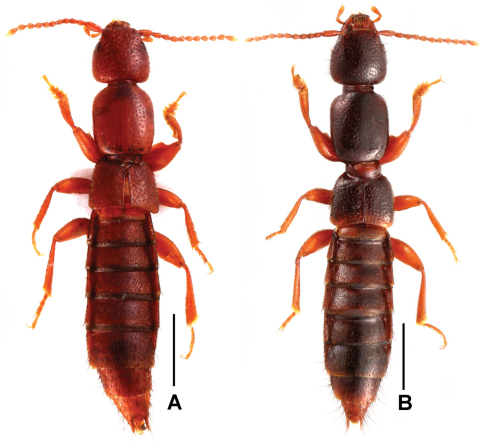
Male habitus of *Lathrobium* spp., **A** *Lathrobium uncum***B** *Lathrobium* sp. indet. Scales: 1.0 mm.

Habitus as in Fig. 2A. Externally similar to *Lathrobium lingae*, except for the lighter average coloration, the somewhat smaller body size and the sparser punctation on the head and the pronotum.

Male. Sternite VII (Fig. 6A) with a group of coarse setae at middle; sternite VIII (Fig. 6B) with half elliptical median emargination, two rows of modified setae in large but faint apical impression and another row along the posterior margin; sternite IX (Fig. 6C) with cuttle-bone-shaped impression; aedeagus (Fig. 6D, 6E) with a hook-shaped ventral process.

Female. Tergite VIII (Fig. 6F) with posterior margin saliently curved; sternite VIII (Fig. 6G) weakly convex posteriorly and with inconspicuous micropubescence; tergite IX (Fig. 6H) (not separated from X), with long lateral processes; tergite X relatively short.

#### Distribution.

East China (Zhejiang: Longwangshan Mountain).

#### Etymology.

The specific name ‘*uncum*’ (Latin adjective) means ‘hooked’.

#### Remarks.

The new species and *Lathrobium tamurai* from Zhejiang share many features, particularly the general form. They can be readily distinguished by the male sternite VII lacking an impression and with several modified discal setae, male sternite VIII with two rows of modified setae in large but faint apical impression and another row along the posterior margin, and slender aedeagus with the ventral process being hook-shaped in *Lathrobium uncum*, whereas in *Lathrobium tamurai*, the male sternite VII has the impression evenly covered with modified setae on each side of the median part; the male sternite VIII has a horseshoe-shaped impression with dense modified setae; and the robust aedeagus has a straight ventral process.

### Lathrobium sp. indet.

Figs 2B 7

#### Material studied

(3 ♂♂, 3 ♀♀). 3 ♂♂, 3 ♀♀, labelled ‘**CHINA:** Zhejiang Prov / Anji County / Longwang Mt. / Qianmutian / 25.v.2009, alt. 1300 m / Yuan, Liu, Feng & Yin leg.’.

#### Description.

Measurements and ratios: BL 6.43–6.65. Holotype: HL 0.78, PL 1.12, PW 0.93, EL 0.74, HL/HW 0.95, HW/PW 0.96, HL/PL 0.74, PL/PW 1.20, EL/PL 0.66.

Habitus as in Fig. 2B. Externally similar to *Lathrobium lingae*, except for the somewhat smaller body size and the more oblong pronotum.

Male. Sternite VII (Fig. 7A) with conspicuously modified setae at weak impression; sternite VIII (Fig. 7B) with approximately elliptic impression and furnished with numerous peg-setae, emargination irregularly shaped; sternite IX (Fig. 7C) anisomerous; aedeagus (Fig. 7D, 7E) with broad ventral process and two apical gracile dorsal sclerites.

Female. Posterior margins of tergite VIII (Fig. 7F) indistinctly asymmetrical and sternite VIII (Fig. 7G) obtusely produced at middle; tergite IX (Fig. 7H) not separated clearly and its lateral processes acute apically; tergite X relatively short.

#### Distribution.

East China (Zhejiang: Longwangshan Mountain).

#### Remarks.

The speciesresembles *Lathrobium rougemonti* Watanabe from Zhejiangby sharing the similar form, male sternite VII with weak impression and male sternite VIII with many dark setae in the large impression, but that of aedeagus typically with two closer dorsal sclerites. In *Lathrobium rougemonti*, aedeagus have two widely separated dorsal sclerites. The original description of *Lathrobium rougemonti* is based on the holotype from West Tianmushan Mountain. The type was not examined, but based on the description and the illustration (habitus, male abdominal apex and aedeagus) provided by Watanabe (1999), there is still doubt whether the population from Longwangshan represents a new species. As intermediate form exists, it will be necessary to study the type material of *Lathrobium rougemonti* for clarification. (Assing pers. comm.)

### A key to the Lathrobium species from Longwang – West Tianmu Mountains

**Table:** 

1	Length of body larger than 9 mm	2
–	Length of body no more than 7 mm	3
2	Relatively slender (Fig. 1C), HL/PL no more than 0.75; male sternite VI (Fig. 5A) lacking tuft of pubescence at concavity; aedeagus (Fig. 5E, 5F) with short ventral process. Posterior margin of female tergite VIII (Fig. 5G) weakly asymmetrical; female sternite VIII (Fig. 5H) distinctly pointed in the middle	*Lathrobium tianmushanense* Watanabe
–	Relatively stout (Fig. 1B), HL/PL more than 0.80; male sternite VI (Fig. 4A) with tuft of pubescence at concavity; aedeagus (Fig. 4E, 4F) with long ventral process. Female unknown	*Lathrobium longwangshanense* sp. n.
3	Light brown (Fig. 2A); male sternite VII (Fig. 6A) with modified discal setae; male sternite VIII (Fig. 6B) with sparse modified setae in shallow impression; aedeagus (Fig. 6D, 6E) elongate and with hook-shaped ventral process. Female sternite VIII (Fig. 6G) with inconspicuous micropubescence posteriorly	*Lathrobium uncum* sp. n.
–	Brown (Fig. 1A); male sternite VII (Fig. 3A) with modified setae at postero-median margin; male sternite VIII (Fig. 3C) with dense modified setae in deep impression; aedeagus (Fig. 3D, 3E) robust and not as above. Female sternite VIII without micropubescence posteriorly (*Lathrobium lingae* female unknown)	4
4	Male sternite VII (Fig. 3A) with deep apico-median impression; male sternite VIII (Fig. 3C) with regular triangular emargination; aedeagus (Fig. 3D, 3E) with single dorsal sclerite	*Lathrobium lingae*lingae sp. n.
–	Male sternite VII with shallow apico-median impression; male sternite VIII with irregular emargination, aedeagus with two dorsal sclerites	*Lathrobium rougemonti* Watanabe.

**Figures 3.**
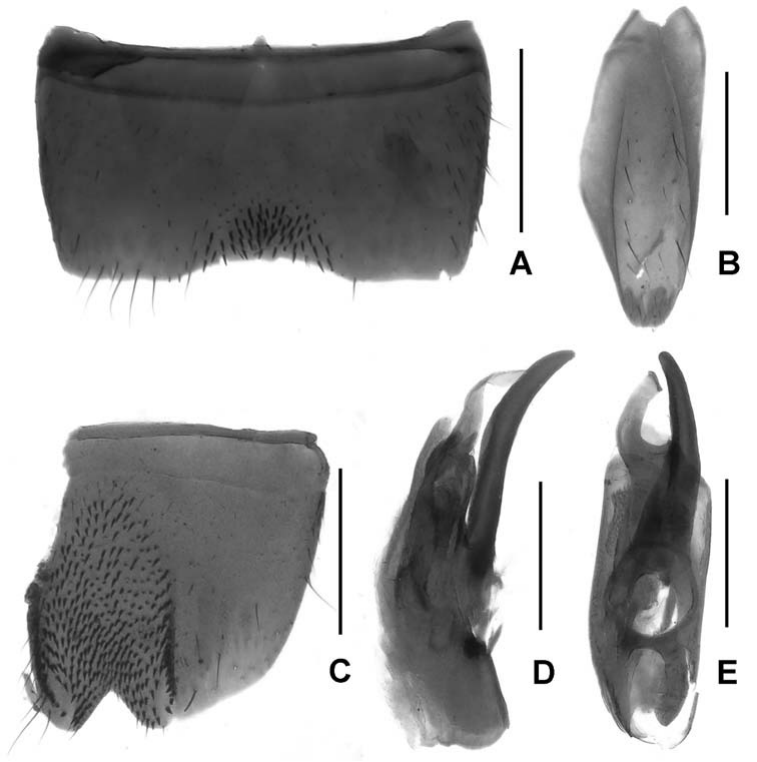
*Lathrobium lingae*. **A** male sternite VII **B** male sternite IX **C** male sternite VIII **D** aedeagus in lateral view **E** aedeagus in ventral view. Scales: 0.5 mm.

**Figures 4.**
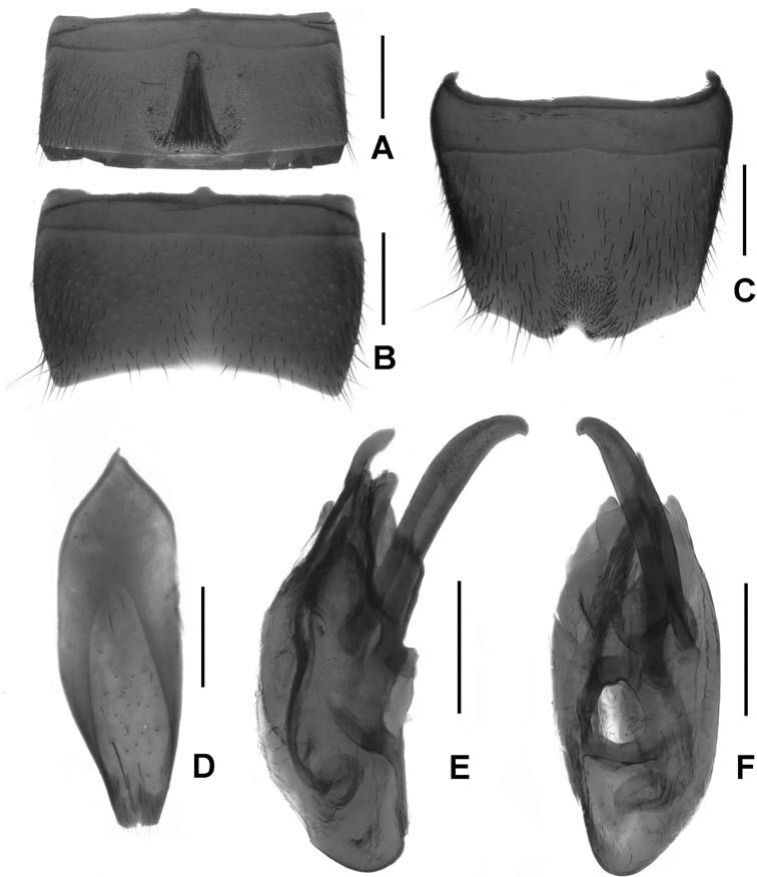
*Lathrobium longwangshanense*. **A** male sternite VI **B** male sternite VII **C** male sternite VIII **D** male sternite IX **E** aedeagus in lateral view **F** aedeagus in ventral view. Scales: 0.5 mm.

**Figures 5.**
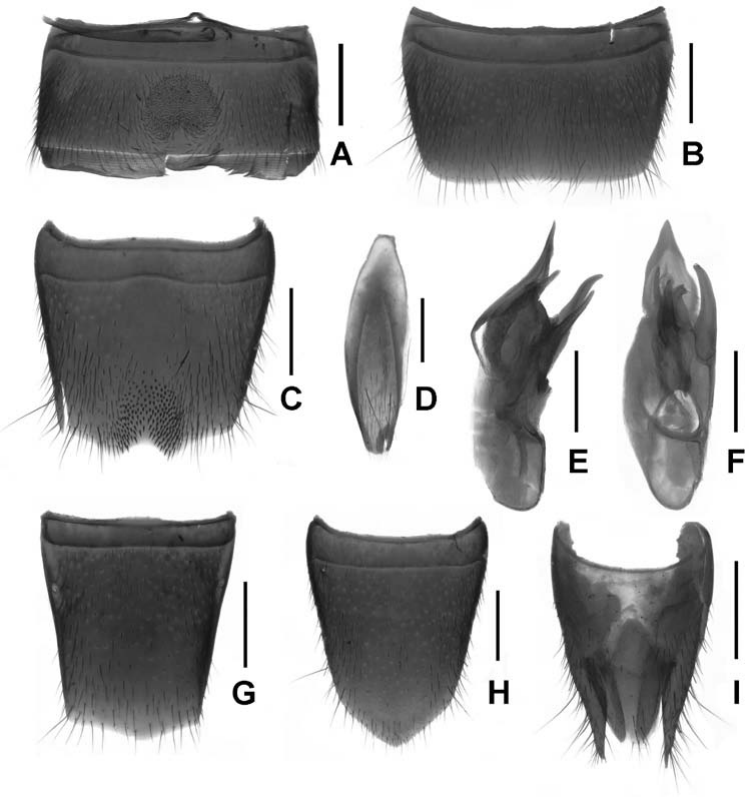
*Lathrobium tianmushanense*. **A** male sternite VI **B** male sternite VII **C** male sternite VIII **D** male sternite IX **E** aedeagus in lateral view **F** aedeagus in ventral view. **G** female tergite VIII**H** female sternite VIII **I** female tergite IX–X. Scales: 0.5 mm.

**Figures 6.**
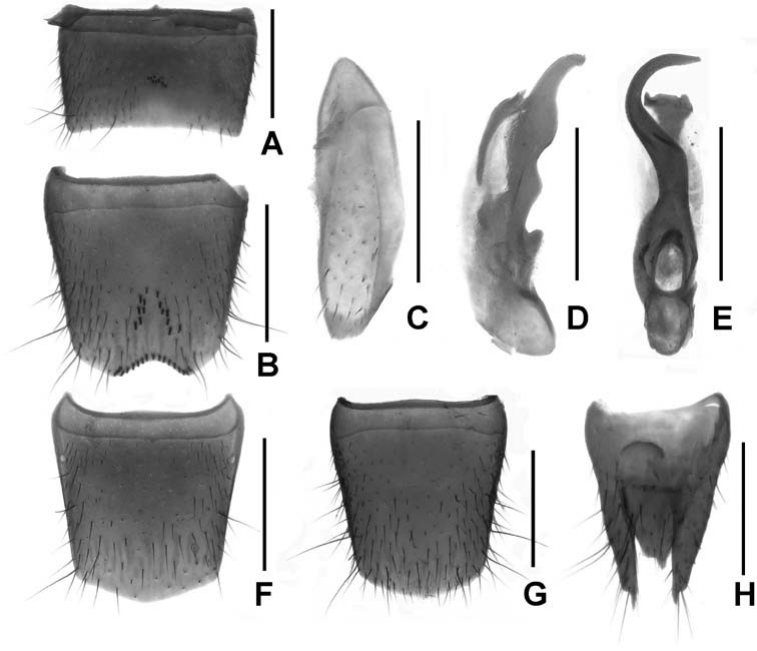
*Lathrobium uncum*. **A** male sternite VII **B** male sternite VIII **C** male sternite IX **D** aedeagus in lateral view **E** aedeagus in ventral view. **F** female tergite VIII**G** female sternite VIII **H** female tergite IX–X. Scales: 0.5 mm.

**Figures 7.**
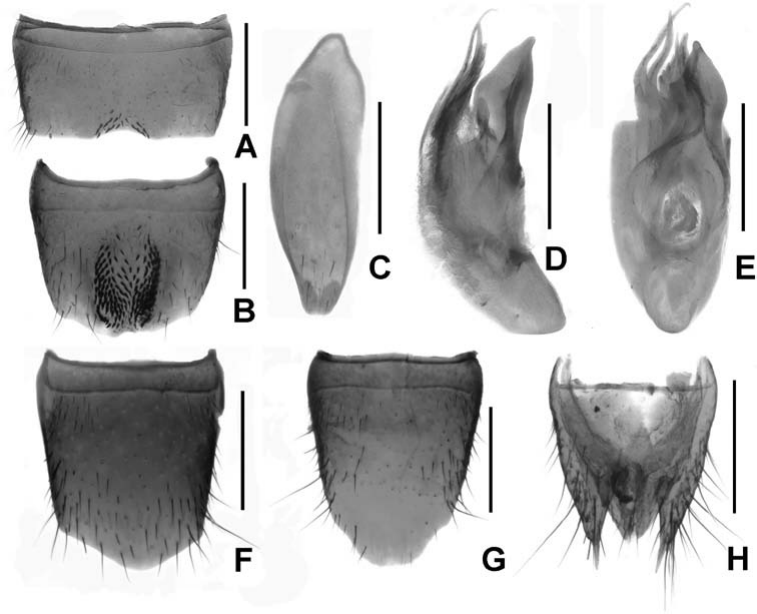
*Lathrobium* sp. indet.. **A** male sternite VII **B** male sternite VIII **C** male sternite IX **D** aedeagus in lateral view **E** aedeagus in ventral view. **F** female tergite VIII**G** female sternite VIII **H** female tergite IX–X. Scales: 0.5 mm.

## Supplementary Material

XML Treatment for Lathrobium lingae

XML Treatment for Lathrobium longwangshanense

XML Treatment for Lathrobium tianmushanense

XML Treatment for Lathrobium uncum

XML Treatment for Lathrobium
